# Bioactive Compounds as Alternative Approaches for Preventing Urinary Tract Infections in the Era of Antibiotic Resistance

**DOI:** 10.3390/antibiotics14020144

**Published:** 2025-02-01

**Authors:** Chiara Cipriani, Marco Carilli, Marta Rizzo, Martino Tony Miele, Paola Sinibaldi-Vallebona, Claudia Matteucci, Pierluigi Bove, Emanuela Balestrieri

**Affiliations:** 1 Department of Experimental Medicine, University of Rome Tor Vergata, Via Montpellier 1, 00133 Rome, Italy; marta.rizzo16@gmail.com (M.R.); miele@med.uniroma2.it (M.T.M.); sinibaldi-vallebona@med.uniroma2.it (P.S.-V.); matteucci@med.uniroma2.it (C.M.); balestrieri@med.uniroma2.it (E.B.); 2 Robotic and Minimally Invasive Urology Unit, Azienda Ospedaliera Universitaria, Policlinico Tor Vergata, Viale Oxford 81, 00133 Rome, Italy; marco.carilli@ptvonline.it (M.C.); pierluigi.bove@uniroma2.it (P.B.); 3 Department of Surgical Sciences, University of Rome Tor Vergata, Via Montpellier 1, 00133 Rome, Italy

**Keywords:** urinary tract infections, non-antibiotic prophylaxis, antibiotic resistance, uropathogens, bioactive compounds, UTI management, natural approach to UTIs, medicinal plant, vitamins

## Abstract

Urinary tract infections (UTIs) are among the most common bacterial infections worldwide. They occur in the urinary system when a microorganism, commonly present on the perineal skin or rectum, reaches the bladder through the urethra, and adheres to the luminal surface of uroepithelial cells, forming biofilms. The treatment of UTIs includes antibiotics, but their indiscriminate use has favored the development of multidrug-resistant bacteria strains, which represent a serious challenge to today’s microbiology. The pathogenesis of the infection and antibiotic resistance synergistically contribute to hindering the eradication of the disease while favoring the establishment of persistent infections. The repeated requirement for antibiotic treatment and the limited therapeutic options have further contributed to the increase in antibiotic resistance and the occurrence of potential relapses by therapeutic failure. To limit antimicrobial resistance and broaden the choice of non-antibiotic preventive approaches, this review reports studies focused on the bacteriostatic/bactericidal activity, inhibition of bacterial adhesion and quorum sensing, restoration of uroepithelial integrity and immune response of molecules, vitamins, and compounds obtained from plants. To date, different supplementations are recommended by the European Association of Urology for the management of UTIs as an alternative approach to antibiotic treatment, while a variety of bioactive compounds are under investigation, mostly at the level of in vitro and preclinical studies. Although the evidence is promising, they are far from being included in the clinical practice of UTIs.

## 1. Urinary Tract Infections and Antibiotic Resistance

Urinary tract infections (UTIs) are among the most common bacterial infections worldwide and typically manifest with symptoms of dysfunction, negatively influencing the patients’ quality of life. The latest guidelines of the European Association of Urology (EAU) group UTIs as uncomplicated and complicated, depending on the absence or presence of underlying health conditions, including anatomical or functional urinary tract abnormalities or comorbidities [1]. Uncomplicated UTIs are usually easy to treat with short-term antibiotics, while complicated UTIs, whose underlying conditions or abnormalities predispose patients to more severe or recurrent infections, require more aggressive and prolonged treatment. Recurrent infections (rUTI) are defined as those that occur with a frequency of at least three infections/year or two infections in the last six months [2] and belong to complicated UTIs. The rUTI can be a relapse of an acute bacterial infection not completely eradicated by drug treatment or a new infection caused by the same microorganism.

Both Gram-positive and Gram-negative bacteria usually cause the infections, although viruses and fungi can also be implicated. *Escherichia coli* (*E. coli*) is the predominant pathogen found in the urine samples of patients with UTIs, although other bacteria such as *Klebsiella pneumonia*, *Proteus mirabilis*, *Enterococcus faecalis*, and *Staphylococcus saprophyticus* can also be present [3,4,5]. Other causes of infections can be bacteria from the gastrointestinal tract or external sources, such as those from sexual activity and catheterization [6]. Uropathogenic strains of *E. coli* (UPEC) are the most commonly encountered bacteria, causing 50% of nosocomial infections and up to 95% of community-acquired urinary tract infections [7]. In the context of hospital-acquired infections, UTIs are the most common healthcare-associated infection, accounting for about 40% of all nosocomial infections. Indeed, catheter-associated UTIs are particularly prevalent, with an incidence rate of about 3–7% per day of catheterization [8,9]. UTIs are prevalent across all age groups but are particularly common in sexually active women, older adults, and children. They occur more frequently in women than in men, with about 50–60% of women experiencing at least one infection in their lifetime. This difference is mainly due to anatomical characteristics. As such, in women, the urethra is located closer to the anus, facilitating the transfer of pathogens from the fecal reservoir to the bladder and eventually to the renal pelvis, and it is also shorter, allowing easier access to the bladder. Additionally, women are more susceptible than men to rUTIs, with studies indicating that up to 25–30% of women who have had one UTI will experience another within six months. Due to physiological changes in the urinary tract, pregnancy is also an additional risk factor [10]. The incidence of infections also increases with age, particularly in postmenopausal women and in men with prostate issues, such as enlargement, urinary obstruction, or catheter use [11]. UTIs are common in children, especially in the first year of life, with boys more commonly affected in the neonatal period and girls in the later stages of growth.

UTIs can occur in any part of the urinary system when a microorganism, commonly present on the perineal skin or rectum, enters through the urethra, reaching the bladder through the actions of specific adhesins. If the host’s immune system fails to eliminate all bacteria, they begin to multiply, producing toxins and enzymes that promote their survival. Colonization of the kidneys can evolve into bacteremia if the pathogen crosses the kidney epithelial barrier [12]. Bacteria reach the bladder and adhere to the luminal surface of uroepithelial cells despite the flushing effect of urine flow [13], resulting also in biofilm formation. This is an important event in urinary tract infections because bacteria (i) can be protected from the effects of antibiotics and phagocytosis by neutrophils; (ii) by infiltrating deeper into the urothelium, facilitate the onset of recurrences [14], and (iii) promote the emergence of antibiotic resistance [15,16].

The primary treatment for bacterial UTIs involves antibiotics. Broad-spectrum antibiotics are often used initially, followed by targeted therapy based on culture results. Complicated UTIs may recur with an increased risk of complications such as kidney damage or sepsis. Close monitoring and follow-up are often necessary to manage the underlying condition and prevent recurrence, and the combination of antibiotic therapy may be necessary to achieve adequate bacterial clearance in complicated urinary tract infections or cases involving multidrug-resistant organisms [1].

Given the high prevalence of bacterial urinary tract infections and considering the changes in antibiotic susceptibility that uropathogens undergo over time due to resistant mechanisms, the recommended drugs require periodical updating [17]. Several resistance mechanisms adopted by uropathogens have been described. The most widespread is the production of extended-spectrum beta-lactamases (ESBLs), responsible for resistance to penicillins and cephalosporins. The spread of this type of resistance has occurred rapidly because most of the genes encoding the production of ESBLs are carried by plasmids, which can be transferred to bacteria belonging to different genera or even species [18]. UPEC strains have developed resistance, especially to the most commonly used antibiotics, such as trimethoprim-sulfamethoxazole (TMP-SMX) and fluoroquinolones (ciprofloxacin and levofloxacin). The uropathogen *K. pneumoniae*, which is frequently responsible for healthcare-associated urinary tract infections, also produces ESBL enzymes and carbapenemases, making treatment of infections difficult. Among the Streptococci involved in urinary tract infections, *Enterococcus faecalis* is often resistant to many antibiotics, including ampicillin and vancomycin [19]. Therefore, antibiotic resistance in UTIs is a growing public health concern that requires a complex approach, including improved antibiotic stewardship, infection control, new treatments, and preventive or combined strategies [20,21].

The World Health Organization (WHO) has identified the need for a change in the strategy in line with the ‘One Health’ approach to address antimicrobial resistance in the contexts of human, animal, and environmental health [22]. Concerning UTIs, antibiotics remain the gold standard for treatment and prevention. Changing the therapeutic strategy by including non-antibiotic approaches could help avoid the emergence of antimicrobial resistance, especially in patients with recurrent infections. Indeed, the repeated request for antibiotic treatments and the limited available therapeutic options have significantly contributed to the increase in antibiotic resistance, creating clinical and economic burdens on healthcare systems [23]. This narrative review aims to contribute to the discussion on changing strategies to address antimicrobial resistance by reviewing the current evidence on non-antibiotic approaches for the management of urinary infections. First, starting with the EAU guidelines, supplements currently used in clinical practice for UTI management will be described. Second, evidence on plant-derived antimicrobial bioactive compounds and vitamins as potential new therapeutic approaches for the treatment of UTIs will be overviewed.

## 2. Supplementations Cited in the European Association of Urology Guidelines for Urinary Tract Infections Management

**Figure 1:**
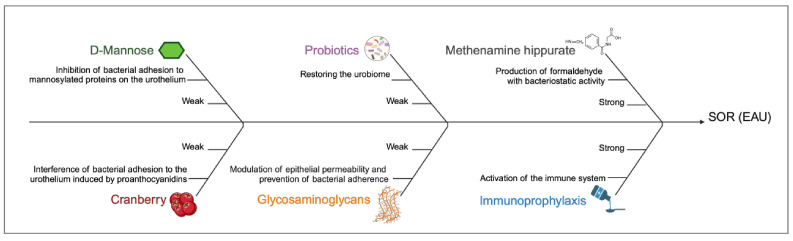
Non-antibiotic approaches for urinary tract infections management recommended by the European Association of Urology guidelines on urological infections. Among non-antibiotic interventions aiming to prevent recurrent urinary tract infections, several supplementations are cited by European Association of Urology (EAU) guidelines with different strength rates of recommendation (SOR). Created in https://BioRender.com (accessed on 27 September 2024).

Despite the leading role of antibiotics in UTI management, either as full-dose treatment for active infections or low-dose prophylaxis for recurrences, the development of antibiotic resistance is a major concern and creates a demand for alternative treatment options. Among non-antibiotic interventions aiming to prevent recurrent UTIs, several supplementations are cited by EAU guidelines (Figure 1). Although they do not interfere directly with resistance mechanisms, these supplements can equally play an important role in the prevention of recurrent infections and counteracting microbial virulence through the enhancement of host defense mechanisms. Therefore, supplementation, by enhancing and supporting the activity of the immune system, may indirectly help mitigate or inhibit the development of antibiotic-resistant bacteria [24]. These agents are generally safe and well-tolerated, making them ideal for chronic intake. Unfortunately, most of them showed conflicting results, which translates into “weak” recommendations for their use [1].

**D-mannose** is a monosaccharide isomer of glucose administered orally and excreted by urine. The suggested mechanism of action in UTI prevention relies on its ability to inhibit the interaction between virulence factors of bacteria, such as the FimH protein domain of the type 1 pili of *E. coli* and mannosylated proteins on the surface of urothelial cells [25] (Figure 1). Preclinical and clinical studies showed that D-mannose has a potential role in UTI management, both for enhancing antibiotic effect when administered in combination during acute infection, as well as non-antibiotic prophylaxis for recurrences when administered alone or combined with other supplementations [26,27]. In published clinical trials, D-mannose as a single active ingredient exhibits efficacy at daily doses between 2 and 3 g. Moreover, no serious adverse event was described, except for bloating and diarrhea at high doses, or hyperglycemia among diabetics [28]. Recently, results from a Cochrane systematic review including 7 RCTs (719 patients) showed that the overall quality of the evidence was low, mainly due to the lack of high-quality trials [29]. For this reason, EAU guidelines recommend informing the patients about the overall weak and contradictory pieces of evidence on its effectiveness (strength rating: “weak”) [1].

**Cranberries** belong to the Ericaceae family; the most widespread species are *Vaccinium oxycoccos* and *V. macrocarpon.* Several compounds have been identified in the berries, but the most relevant for their clinical application are the extracted proanthocyanidins (PAC) and polyphenols, which play a role in the natural plant defense system against microbial infections [30]. Several mechanisms of action have been proposed to explain the properties of cranberries in recurrent UTI prevention. To date, the interference of bacterial adhesion with urothelium induced by PAC seems to be the best candidate (Figure 1). PAC in cranberries are characterized by a specific structural feature (A-type linkages, i.e., the double bond between oligomers and polymers of flavans), which is similar in structure to the bacterial-binding receptor on urothelial cells (P fimbriae of *E. coli*) [31]. Cranberry can be administered in several forms (e.g., whole berries, tablets, juices, etc.), and the PAC concentration could depend on the administration form. Indeed, in the majority of clinical trials, PAC concentrations are not disclosed, adding heterogeneity and difficulty in interpreting results. According to ex vivo studies, a significant bacterial anti-adhesion activity can be identified with at least 36 mg/day of PAC [32]. However, a recent Randomized Controlled Trial (RCT) of 145 women comparing high- versus low-dose cranberry PAC extract reported no significant differences between groups in terms of symptomatic UTI reduction [33]. Currently, the quality of evidence regarding the efficacy of cranberry products in UTI prevention is low, and several meta-analyses have failed to draw any definitive conclusion [34,35]. However, a subsequent meta-analysis showed that cranberry products significantly reduced the incidence of UTI, particularly in individuals with recurrent infections [36]. Notwithstanding these conflicting results, cranberry products have several advantages (cheapness, high safety profile, no risk of bacterial resistance), so according to EAU guidelines, their use could be suggested to well-informed patients (strength rating: “weak”) [1].

**Probiotics** are defined as living microorganisms that, when administered in adequate amounts, confer a health benefit on the host, maintaining the homeostasis of the microbiome [37]. Historically, the urinary tract was thought to be a sterile environment; in the last decade, the existence of a urinary microbiome (urobiome) has been acknowledged, and several studies have demonstrated how imbalances in the microbial community potentially contribute to UTI pathogenesis [38]. Moreover, it has been hypothesized that vaginal and gut microbiomes can also play a role in UTI recurrences (for example, harboring uropathogenic reservoirs) [39]. The male urobiome, where *Corynebacterium* has been identified as the dominant genus, seems to resemble those present in the skin microbiome, while the female urobiome demonstrates several similarities to the vaginal microbiome (*Lactobacillus* species are predominant) [40]. *Lactobacilli* are thought to prevent UTIs through several mechanisms, including (1) inhibition of uropathogenic adhesion to the vaginal epithelium (by exclusion, competition, or displacement); (2) production of bacteriocins (i.e., peptides produced by bacteria to kill competing organisms) and hydrogen peroxide (inducing membrane distress); (3) acidic environment caused by lactic acid; (4) inhibition of bacterial biofilm formation; and (5) downregulation of pro-inflammatory cytokines (TNF, IL-6, etc.) [41]. The normal microbiome can be altered in the presence of some conditions, such as an indwelling catheter or other devices, age, low estrogen, diabetes, neurogenic lower urinary tract disorders, prolonged antibiotic therapies, etc. Those risk factors help the proliferation of uropathogens, leading to UTI development [42].

Restoring the normal urobiome with probiotics is an interesting/attractive strategy for recurrent UTIs (Figure 1). Probiotics can be administered orally or vaginally, but no recommendation can be made in terms of dosage or treatment duration. Moreover, major clinical benefits have been described only for certain strains of *Lactobacilli* (e.g., *L. rhamnosus* GR-1, *L. reuteri* RC-14, *L. crispatus* CTV-05) [43,44]. From a pre-clinical standpoint, not all *Lactobacillus* strains showed similar efficacy against uropathogens: for example, *L. crispatus* showed a greater capacity to block uropathogen adherence [45]. In addition, although all species produce organic acids, not all produce hydrogen peroxide and bacteriocin [46]. The conflicting results from clinical trials depend on several factors, including *lactobacillus* strains used, route of delivery, dosage, duration, and population studied (for example, age and race can influence normal vaginal microbiome composition). For these reasons, it is very difficult to address which specific regimen is best. Daily oral administration of *L. rhamnosus* GR-1 and *L. reuteri* RC-14 in healthy women without previous history of UTIs led to a significant increase in vaginal *lactobacilli* after two months of treatment compared to the control group [47]. Another RCT in postmenopausal women with recurrent UTIs showed an efficacy of the same *lactobacillus* strains in UTI prevention, although this effect was lower than that obtained by antibiotic prophylaxis with trimethoprim-sulfamethoxazole [44]. Finally, an RCT in randomly assigned premenopausal women, using a vaginal suppository containing *L. crispatus* once daily for five days and then weekly for 10 weeks or a placebo, showed a significant reduction in UTI episodes in the treated group, with increased vaginal colonization with *L. crispatus* during follow-up [43]. Results from a Cochrane systematic review of nine studies (735 patients) concluded that probiotics did not provide any significant benefit compared to placebo or no treatment in UTI prevention [48]. However, the risk of bias was high, mainly due to the heterogeneity of studies included in the analysis. To date, there are no active phase III trials investigating the role of probiotics in recurrent UTI prophylaxis. Consequently, taking into account the low quality of available data, EAU guidelines recommend the use of probiotics containing strains of proven efficacy to prevent UTIs (strength rating: “weak”) [1].

**Glycosaminoglycans** (GAGs) are abundantly present on the surface of the bladder urothelium where, together with other highly negatively charged polysaccharides, they constitute the mucous layer. It has been hypothesized that this “coating” may play an important role in modulating epithelial permeability and preventing bacterial adherence [49,50]. Moreover, preclinical and clinical findings suggest that loss of the GAGs layer in the bladder could be the initial step towards several chronic diseases (e.g., interstitial cystitis, radiation cystitis, overactive bladder, recurrent UTI) [51] (Figure 1). Taking into account these points, EAU guidelines recommend endovesical installations of GAGs in patients with recurrent UTI where less invasive preventive approaches have been unsuccessful (strength rating: “weak”) [1,52]. Results from a meta-analysis of two RCTs and six non-RCTs (800 patients) showed that endovesical instillation of GAGs reduces the rate of UTI and increases the time to recurrence in women with recurrent UTI; furthermore, GAG treatment was associated with an improvement in patient-reported outcomes [53]. Indeed, endovesical instillations might not be considered a “supplementation”, but rather a treatment (also given its invasiveness). For this reason, several companies have developed oral formulations of GAGs for managing recurrent UTIs. To date, we have only a few reports on this topic, highlighting promising results [54,55]. Considering the paucity of literature data, this route of administration is still not included in international guidelines on UTI management.

**Methenamine hippurate** (MH) is a salt whose hydrolysis in an acid environment (urine pH < 5.5) leads to the production of formaldehyde, which has bacteriostatic activity, inhibiting cell division (Figure 1). Moreover, the formation of 1,3-thiazane-4-carboxylic acid blocks the synthesis of methionine, an essential amino acid involved in cytoplasmic synthesis [56]. Since formaldehyde has non-specific bactericidal activity, it does not induce the development of antimicrobial resistance. Methenamine salts are well tolerated, and adverse effects are generally mild. The recommended dosing regimen is 1 g twice daily [57]. Results from a Cochrane systematic review of 13 studies (2032 patients), with high levels of heterogeneity, showed that MH may be effective for preventing UTI in patients without renal tract abnormalities, particularly when used for short-term prophylaxis [58]. Moreover, a recent non-inferiority RCT of 240 women comparing twice-daily MH to once-daily low-dose antibiotic prophylaxis for 12 months concluded that both regimens had similar incidence rates of symptomatic UTIs [59].

Based on these results, MH use is recommended by EAU guidelines to reduce recurrent UTI episodes (strength rating: “strong”) [1].

**Immunoprophylaxis** is based on the activation of the immune system induced by several inactivated uropathogens that produce bacteria-specific antibodies [60]. In this regard, several vaccines have been studied for UTI prophylaxis (Figure 1).

Uro-Vaxom is an oral vaccine containing 18 serotypes of uropathogenic *E. coli* (UPEC) obtained by heat-killing (OM-89) or lysis (OM-89-S). Several meta-analyses and systematic reviews based on nine RCTs showed that OM-89 is effective in recurrent UTI prevention compared to placebo at short-term follow-up (<6 months) [44,61,62,63]. However, another trial of 451 patients did not demonstrate a preventive effect of OM-89-S compared to placebo [64]. Finally, it is important to underline that Uro-Vaxom is active only against *E. coli*, since it contains only strains of UPEC.

Other vaccines which showed interesting results in terms of recurrent UTI prevention (at least at short-term follow-up) are the Solco-Urovac (a vaginal vaccine containing 10 strains of heat-killed uropathogens such as UPEC, *P. mirabilis*, *Morganella morganii*, *K. pneumoniae*, and *E. faecalis*) and the Uromune (or MV140, a sublingual spray containing whole-cell inactivated *E. coli*, *K. pneumoniae*, *P. vulgaris*, and *E. faecalis*) [63]. In particular, MV140 was evaluated in a multicenter, randomized, double-blind, placebo-controlled study on 240 women, showing a significant reduction in UTI episodes at 12 months follow-up [65].

These findings led EAU guidelines to recommend immunoprophylaxis use in adults with recurrent UTI (strength rating: “strong”) [1].

## 3. Promising Roles of Medical Plants and Vitamins in Urinary Tract Infection Management

### 3.1. Medicinal Plants

**Figure 2:**
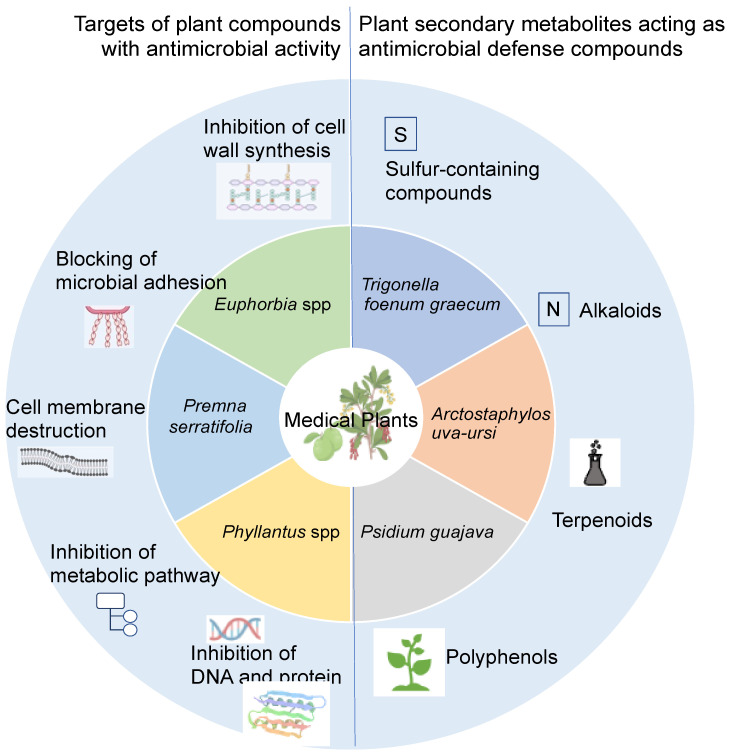
**Medicinal plants as potential sources for urinary tract infection management.** Plants used for medicinal purposes have various antimicrobial defense mechanisms, producing natural compounds (secondary metabolites) that act as natural antibiotics against pathogenic microorganisms. Created in https://BioRender.com. (accessed on 27 September 2024).

Traditional medicine encompasses a wide range of knowledge, skills, and practices derived from different cultures. For millennia, medicinal plants have been the basis of medicine and continue to represent a potential source of new bioactive compounds [66,67]. Plants used for medicinal purposes have various antimicrobial mechanisms, producing natural compounds that act as natural antibiotics against pathogenic microorganisms [68]. Based on their chemical structure, these secondary metabolites are classified into sulfur-containing compounds, alkaloids, terpenoids, and polyphenols, which act on different target sites by inhibition of cell wall biosynthesis, microbial adhesion to epithelial cells, cell membrane disruption, inhibition of metabolic pathways, and DNA replication and repair (Figure 2) [69].

However, to date, their mechanisms of action have not been fully elucidated. Although some plant derivatives have already been approved for therapeutic use, studies are still ongoing to identify further active principles, allowing a wider choice for treatment, especially for recurrent infections [70,71,72]. Interestingly, one of the most abundant sources of medicinal plants is the island of Guam, located in the tropical area of the western Pacific [73]. About 200 medicinal herbs and plants grow on the island, which have been used for centuries as drugs and food supplements for their recognized activity in preventing numerous diseases. Urinary tract infections are among the diseases that local healers traditionally treat with these herbs [74,75,76,77].

***Arctostaphylos uva-ursi*** is a plant whose leaves are used to obtain extracts traditionally recommended for inducing diuresis and for the treatment of UTIs, although the efficacy and any adverse effects have not been documented [78,79]. The active component of the extract is arbutin, a hydroquinone glycoside, which accounts for 5–16% of dried *Arctostaphylos uva-ursi* leaves [80]. Arbutin is absorbed from the gastrointestinal tract, metabolized to glucuronide in the liver, and hydrolyzed during renal excretion to form the active compound, hydroquinone, which is believed to have local antiseptic activity on the urinary mucous membrane [81,82]. Although *Arctostaphylos uva-ursi* represents a safe therapeutic option for the treatment of lower urinary tract infections, it is important to note that hydroquinone is a potential carcinogen and therefore, it should not be taken chronically [83]. Of note, *Arctostaphylos uva-ursi* leaf extracts may cause side effects, including nausea, vomiting, irritability, and insomnia, and it is not recommended for pregnant or lactating women [84,85,86,87].

A randomized trial was conducted on 382 women aged 18–70 years with urinary tract symptoms suggestive of infection, who were treated with ibuprofen or *uva-ursi*, with a combination of both, or with placebo for 3–5 days before antibiotics treatment. The analysis showed that after 2–4 days of treatment, the urinary symptoms were comparable in the four groups considered, and the outcome of subsequent antibiotic treatment was not affected in any way by the intake of *Arctostaphylos uva-ursi*. Of note, only the recommendation to delay treatment had a positive outcome of reduced use of antibiotics [88]. A double-blind randomized controlled trial was conducted including adult women with suspected uncomplicated UTIs receiving either *Arctostaphylos uva-ursi* as tablets for 5 days or fosfomycin as 3 g single dose, and their respective placebos. The obtained results demonstrated that initial treatment with *uva-ursi* reduced antibiotic use but led to a higher symptom burden and more safety concerns than fosfomycin [89].

Other compounds have also been extracted from dried *Arctostaphylos uva-ursi* leaves, including tannins (up to 30%), flavonoids (up to 1.5%), and triterpenes (0.4–0.8%) [81]. Tannins have been shown to act on Gram-positive and Gram-negative bacteria replication, inhibiting cell wall synthesis, disrupting the cell membrane, altering fatty acid biosynthetic pathways [90,91,92,93,94], and acting as quorum sensing inhibitors [95,96]. Tannin-loaded nanoparticles/hydrogels have been tested for their antibacterial effects to reduce the dosage and toxicity of natural products [97] while maintaining and/or enhancing their efficacy. The antibacterial efficacy of tannins has also been evaluated in clinical studies comparing a cranberry mouthwash with one containing chlorhexidine, showing that both inhibited the bacterial load of *Streptococcus mutans* by 68% [98]. *Arctostaphylos uva-ursi* dried leaf extracts contain numerous structurally different bioactive compounds, the action of which has not yet been fully elucidated. Therefore, it should be taken into account that this complexity could be a risk for patients due to potential herb-drug interactions, likely resulting in synergistic, additive, and/or antagonistic effects [99,100].

***Euphorbia* spp.** have numerous uses in ethnic and traditional medicine. In particular, *E. hirta* dried herb decoction is used to treat skin diseases, the fresh herb decoction is used to treat thrush, the root decoction increases milk production in women, while the roots are used to treat snakebites. In addition, the aqueous extract of *E. hirta* leaves was traditionally used as a diuretic agent in East Africa [101,102]. In vitro studies demonstrated the efficacy of *E. hirta* against *E. coli*, *Salmonella typhi*, *K. pneumonia*, *P. vulgaris*, and *Pseudomonas aeruginosa*, suggesting its possible use in the treatment of UTIs and typhoid fever [103,104]. The antimicrobial activity of *E. hirta* leaf extract, evaluated in vitro [105], was found to be mainly dependent on the diluent used. Decoction suppressed the growth of *Staphylococcus aureus* and *P. aeruginosa* [106], while methanolic and ethanol extract had the strongest antibacterial activity against *S. aureus*, *P. aeruginosa*, and *E. coli*, compared to acetone, ethyl acetate, and hot aqueous extracts [107,108,109].

***Euphorbia lateriflora*** is used in ethnomedicine for treating several conditions, including hematological disorders, venereal diseases, subcutaneous parasitic infections, and UTIs [110,111]. A recent in vitro study evaluated the activity of extracts and fractions of *E. lateriflora* on bacterial and fungal isolates showing multidrug resistance obtained from patients who suffered from symptomatic urinary tract infections and vaginosis. *E. lateriflora* exhibited antimicrobial activity that could be due to the presence of saponins, tannins, and flavonoids [112].

***Phyllanthus* spp.** (family Euphorbiaceae) and in particular, *P. amarus* is widely used in traditional Indian medicine for the treatment of diabetes and for its antiviral, antibacterial, anti-inflammatory, antimalarial, antitumor, antioxidant, hepatoprotective, nephroprotective, and diuretic properties [113]. In vitro studies have shown that the methanolic extract of the aerial portion of the plant has antibacterial activity against most microorganisms responsible for urinary tract infections, such as *S. aureus*, *Serratia marcescens*, *E. coli*, *Enterobacter* spp., *Streptococcus faecalis*, *K. pneumoniae*, *P. mirabilis*, and *P. aeruginosa* while, extracts obtained with other solvents, showed reduced activity [114,115,116].

As *P. amarus*, fruits, flowers, seeds, leaves and bark of the plant *Phyllanthus emblica* also have been used to obtain numerous remedies used in traditional medicine. In vitro and preclinical studies have shown that *P. emblica* has antioxidant, antitumor, immunomodulatory, antiviral, anti-dyslipidemic, anti-apoptotic, anti-inflammatory, hepato- and nephroprotective, and antidiabetic properties [117]. In vitro studies have also shown that the extracts possess different antimicrobial properties against several Gram-positive and negative bacteria (*Bacillus subtilis*, *S. aureus*, *S. dysenteriae*, *P. aeruginosa*, antimicrobial-resistant *S. Typhi* and *S. Enteritidis*), depending on the type of solvent used in the extraction procedure [118,119,120].

***Psidium guajava***, a tropical food plant belonging to the Myrtaceae family, commonly known as guava, has been shown to possess numerous properties that have been widely used to treat several pathologies [121,122,123].

Several substances with beneficial effects can be extracted from different parts of the plant, such as the leaves, bark, roots, seeds, and fruit, due to their rich nutritional and phytochemical profile [124,125]. Recent studies have shown that bioactive phytochemicals obtained also exhibit anti-tumor activity [126].

In particular, the leaves have been used in folk medicine as a traditional herbal remedy for the treatment of bladder infections, vaginal douches, and wound disinfection, but also for gastroenteritis treatment [74,127,128]. In 2008, Pelegrini et al. [129] isolated an antimicrobial peptide from guava seeds found to be active against urinary and gastrointestinal pathogens, such as *P. mirabilis*, *K. pneumonia*, and *E. coli* strains, obtained from hospitalized patients. Amino acid sequencing revealed a glycine-rich protein active against Gram-negative bacteria with 3D structural homology to an enterotoxin derived from *E. coli*, suggesting it may act via dimer formation. Subsequently, a recombinant peptide synthesized in *E. coli* by Tavares et al. demonstrated that guava seeds contain peptides with inhibitory activity against Gram-negative and Gram-positive pathogenic bacteria and polyphenols responsible for bacteriostatic or bactericidal activity [130]. Alcoholic and aqueous extracts of *P. guajva* leaves and stems have also shown antibacterial activity against *S. aureus* isolates obtained from patients with urinary tract infections. These antibacterial effects have been potentially attributed to polyphenols, which can interact with bacterial cell membranes [131]. Indeed, it has been shown that *P. guajava* is naturally rich in bioactive compounds, including ascorbic acid (vitamin C), phenols, carotenoids, dietary fiber, and minerals [132].

Recently, a study was conducted to determine in vitro the susceptibility and synergistic properties of the antimicrobial activity of the aqueous extract of guava leaves and the antimicrobial drugs most frequently used to treat UPEC infections. The evaluation, performed by antibiogram on 180 urine samples from symptomatic patients, showed that guava extract increased the efficacy of antibiotics commonly used for the treatment of UTIs, in particular of the antibiotic ofloxacin, suggesting that the antibiotic-guava extract combination may help to delay the onset of bacterial resistance [133].

***Premna serratifolia***, family Lamiaceae, is traditionally used to treat cough, asthma, ear infections, hemorrhages, and urinary tract disorders [73,74]. Among the compounds identified in the ethanol extract of *P. serratifolia* leaves [134], forsythoside B was found to have antimicrobial activity against *P. mirabilis* and *S. aureus*, including one methicillin-resistant strain [135]. At the same time, campneoside was shown to have efficacy against *S. aureus*, *Streptococcus pyogenes*, and *Streptococcus faecium* [136]. Recently, forsythoside A, a compound also present in *P. serratifolia* leaf extract, was found to be active against lipopolysaccharides by inhibiting the TLR4 signaling pathway, both in vitro and in animal studies. Therefore, its use has been suggested as a possible new treatment for LPS-induced diseases [137].

***Trigonella foenum graecum* (Fenugreek)**, a plant of the Fabaceae family, has been used since the time of the ancient Egyptians to treat menopause-related disorders, digestive problems, and to induce childbirth. The antibacterial activity of fenugreek has been evaluated in both in vitro and in vivo studies against microorganisms present in the oral cavity, demonstrating that the phytoextract possesses anti-inflammatory properties and antimicrobial activity, in particular against *Streptococcus mutants* [138,139]. In vitro studies have shown that the antimicrobial activity of fenugreek extracts against Gram-negative and positive bacteria depends on the solvent used for the preparation [140,141]. Recently, research has focused on the anti-quorum sensing properties of sotolon [142,143], the aromatic compound that gives fenugreek its typical aroma and flavor, derived from furanone (3-hydroxy-4,5-dimethyl-2(5H)-furanone) [144]. The anti-QS activity of sotolon has been evaluated on *Pseudomonas aeruginosa* using a sub-minimum inhibitory concentration. Sotolon has been demonstrated to reduce the production of bacterial biofilm and virulence factors, the expression of QS genes, and to protect animals from *P. aeruginosa*, suggesting sotolon may be used in the treatment of bacterial infections as an alternative or adjuvant to antibiotics [142].

### 3.2. Vitamins

**Figure 3:**
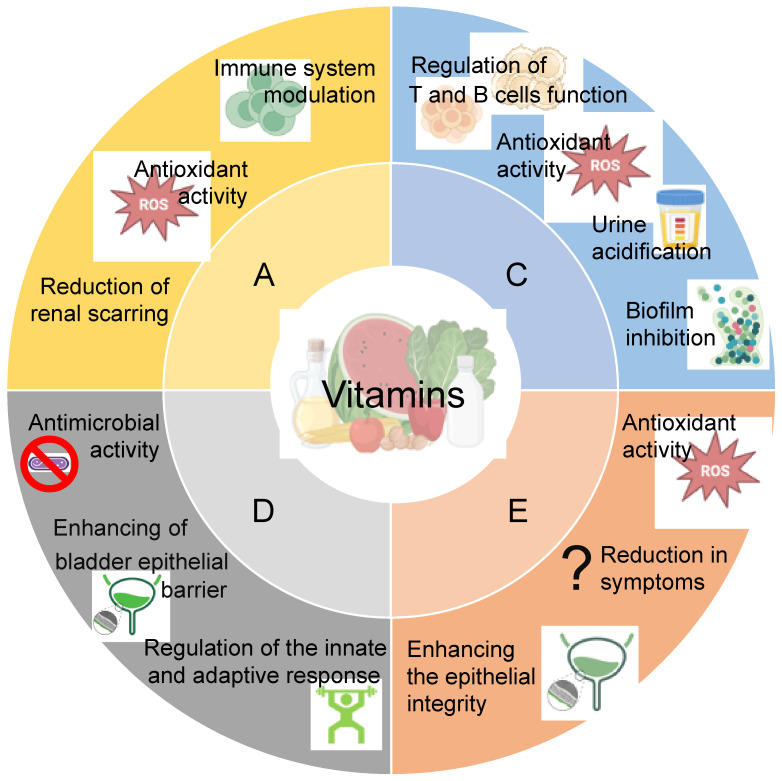
**Vitamins supplementation for urinary tract infection prevention.** Vitamins (A, C, D, and E) show different activities comprising antimicrobial activity, modulation of the immune response, antioxidant properties, and enhancing epithelial integrity. Created in https://BioRender.com. (accessed on 27 September 2024).

Among the available supplements, vitamins are of significant importance as essential nutrients for the maintenance of human health. To date, their therapeutic potential often goes unnoticed, even though vitamins can be used as a support for the treatment and prevention of diseases, in both adults and children (Figure 3) [145].

**Vitamin D** is a fat-soluble vitamin already present in some foods, added to others, and available as a dietary supplement. It is obtained only to a minimal extent from food, but exposure of the skin to the sun is the main source of its production in its active form. Regardless of the source, vitamin D is biologically inert and is activated by two successive hydroxylation processes, the first in the liver and the second in the kidneys. One-third of the world’s population suffers from vitamin D hypovitaminosis, mainly due to inadequate exposure to sunlight [146]. Indeed, vitamin D is essential for human health, as it acts as a hormone that affects the intestinal process of calcium and phosphorus adsorption, and also contributes to the regulation of physiological processes such as bone mineralization. Vitamin D is also required for skeletal growth and remodeling by osteoblasts and osteoclasts. An adequate plasma concentration of vitamin D also prevents rickets in children and, together with calcium, helps protect the elderly from osteoporosis [145,147]. Vitamin D deficiency seems to be associated with the risk of many chronic illnesses, including cancers, cardiovascular disease, and autoimmune and infectious diseases [148,149,150,151,152]. Regarding UTIs, Vitamin D deficiency has been recognized as a risk factor in women of reproductive age and children but not in men [153,154,155,156,157,158,159]. Furthermore, the Vitamin D receptor gene polymorphism is also an important factor for UTI susceptibility, at least in children [160].

In an RCT, 511 subjects with prediabetes were randomized to vitamin D3 (20,000 IU per week) versus placebo for five years. A questionnaire on respiratory tract infections and UTIs was completed every six months, highlighting that vitamin D supplementation could prevent UTIs [161]. However, a randomized triple-blind placebo-controlled clinical trial conducted on 68 children and adolescents with rUTI showed opposite results. As such, patients were randomly assigned to two groups and received either vitamin D (1000 IU/daily) or a placebo for six months, and the serum concentration of vitamin D before and after the study. The frequency of UTIs during the study was recorded. The findings of this trial revealed that vitamin D supplementation has no significant impact on preventing rUTI, likely due to the dose administered [162].

Recently, a network meta-analysis of 50 randomized controlled trials, comprising 10,495 subjects grouped by age, analyzed the impact of administration of D-mannose, vaccine, probiotics, cranberry, and vitamins A and D, alone or in several combinations. The analysis demonstrated that the administration of D-mannose, vaccine, probiotics, cranberry, and the association of cranberry plus probiotics plus vitamin A exhibited a significant reduction in UTI incidence compared to the placebo. Probiotics showed their maximum efficacy in the non-adult group, while vitamin D showed the maximum efficacy in the long follow-up group [24]. The association between vitamin D deficiency and UTIs may be mainly related to a non-classical action of vitamin D in regulating the immune response, both innate and adaptive [163]. Antigen-presenting cells, including macrophages and dendritic cells, T cells, and B cells, can synthesize and respond to vitamin D, as they express 1-α-hydroxylase (that allows the second hydroxylation) to activate the vitamin D and the functional vitamin D receptor (VDR) signaling pathway [164]. Over the course of an infection, VDR signaling modulates innate immune signals through the production of genes encoding antimicrobial proteins (AMPs), including cathelicidins, defensins, hepcidins, and neutrophil peptides [165]. AMPs act as intrinsic antibiotics due to their direct antimicrobial activity against various pathogens [166]. Therefore, in the context of infections sustained by antibiotic-resistant pathogens, such host defense peptides could be valuable candidates for the development of alternative therapeutic agents [167]. In particular, in UTIs caused by *E. coli*, vitamin D may also improve the strength of the bladder epithelial barrier by inducing the expression of the tight junction proteins, thus reducing bacterial invasion [168].

**Vitamin A**, like vitamin D, is a fat-soluble vitamin, important for vision and essential for embryonic development, as it is required for tissue growth and differentiation, cell division, reproduction, and immunity. It is also known for its antioxidant properties. Vitamin A has a crucial role in gene regulation through all-trans and 9-cis retinoic acids that bind and activate their nuclear receptors (retinoic acid receptors) to regulate target genes [169]. Its deficiency has been associated with reduced resistance to infectious diseases and increased risk of immune system over-reaction, chronic inflammation, stronger allergic reactions, and autoimmune diseases, modulating the T cell function and acting as an element with antioxidant and anti-inflammatory effects [170,171,172,173]. Vitamin A has been shown to minimize renal scarring associated with UTIs in rat models, given its ability to promote tissue regeneration [174]. However, this effect has not been confirmed by other studies, which have instead shown tissue damage associated with high doses of vitamin A [175]. Also, in humans, vitamin A appears to have a protective role in children with acute pyelonephritis by reducing renal scarring [176,177,178] and the frequency of recurrence in children with recurrent UTIs [179,180]. Other studies have evaluated combined interventions (vitamin A, probiotics, cranberry, nasturtium, horseradish, and vitamin A in combination with cranberry), demonstrating a reduction in the incidence of UTIs generally with favorable results [24,180].

**Vitamin C** (ascorbic acid) is a water-soluble essential vitamin that cannot be synthesized by the body, so it must be supplemented daily through the diet. Vitamin C is a nutrient required for several important biological functions, primarily as an antioxidant, thereby protecting cellular biomolecules from oxidative damage [181]. It is a cofactor for several enzymes, including those involved in the synthesis of catecholamine hormones [182,183]. Vitamin C plays an important role in the stabilization of the structure of collagen [184], and acts in the regulation of gene transcription and cell signaling pathways [185]. Moreover, it is crucial in controlling the activity of the immune system at various levels, including supporting the integrity of natural barriers and in T and B cell function [186]. Due to its activity as a urine acidifier, Vitamin C could be recommended as a prophylactic agent for the prevention of recurrent urinary tract infections, contributing to creating an unfavorable environment for bacteria growth. Several reports have investigated this aspect but with controversial results. Both in vitro and animal model studies have reported that vitamin C has antibacterial and anti-biofilm formation activity, inhibiting quorum sensing and other regulatory mechanisms underpinning biofilm development, both when used alone or in combination with antibiotics [187,188,189,190,191,192,193]. Even in humans, several studies have highlighted the efficacy of Vitamin C supplementation in treating respiratory tract infections and UTIs, although no definitive evidence has been found on its real long-term efficacy [36,194,195,196,197]. Of note, in a pilot study, women with recurrent UTIs were treated with cranberries, *Lactobacillus rhamnosus*, and vitamin C three times daily for 20 consecutive days monthly for three months and evaluated with a t-structured interview and urinalysis at three and six months following the end of the administration of these supplements. In this setting, no major side effects were recorded, and the administration seemed to represent a safe and effective option for women with rUTIs [198]. In a more recent randomized crossover pilot trial, subjects with rUTI were randomized 1:1 in 2 groups: intervention and control. All participants had an oral preparation of cranberry, D-mannose, propolis extract, turmeric, and Boswellia twice a day for three months. The intervention group also included an oral preparation of hyaluronic acid, chondroitin sulfate, N-acetylglucosamine, and vitamin C once a day for three months. Crossover of treatment occurred at three months for an additional three months. At baseline, three, and six months, participants were evaluated clinically and by standardized questionnaires for urinary symptoms. The results demonstrated that in participants with rUTIs the combination treatment with hyaluronic acid, chondroitin sulfate, N-acetylglucosamine, and vitamin C is effective if started immediately or even a few months after symptoms in participants with rUTI [55].

**Vitamin E** denotes a group of fat-soluble compounds characterized by antioxidant properties and the ability to fight free radicals, inhibiting the production of reactive oxygen species [199]. Vitamin E also plays an important role in cell membrane organization [200] and in the regulation of platelet aggregation [201]. It also has immunomodulatory effects, both by direct activity protecting membrane integrity and indirectly regulating inflammatory factors, affecting the host’s susceptibility to infections [202]. It accumulates in the liver and does not need to be regularly taken through food as the body releases it in small doses when its use is necessary. A study conducted on a rat animal model demonstrated the ability of vitamin E to increase cell-cell adhesion in the uroepithelium, thus restoring the epithelial integrity of the bladder [203]. This evidence supports the possible use of vitamin E as an adjuvant in urinary tract infections treatment. Only one study, conducted on young women affected by acute pyelonephritis, showed a reduction in symptoms (fever, urinary urgency and frequency, and urinary incontinence) at the first episode in patients for whom antibiotic treatment was associated with vitamin E supplementation, compared to those treated with antibiotics alone [204].

## 4. Conclusions

Antibiotic resistance is an emerging threat that involves not only humans but also animals and the environment. In recent decades, the massive and inappropriate use of antibiotics and their spread in the environment have generated widespread evolutionary pressure that has favored the emergence of multidrug-resistant microbial strains. Therefore, in view of a change of strategy to combat antimicrobial resistance, new bioactive compounds have been investigated. In this direction, the EAU guidelines already recommend several supplementations as non-antibiotic interventions to prevent recurrent UTIs. Of note, although these agents are generally safe and well-tolerated, making them ideal for chronic intake, most of them have shown conflicting results, which translates into weak recommendations for their use in clinical practice. In an attempt to contribute to the discussion on non-antibiotic approaches for UTI management, traditional medicine provides insights regarding the antimicrobial activity of plant-derived bioactive compounds. As such, numerous in vitro and animal model studies have demonstrated that plant-derived bioactive compounds have bacteriostatic and/or bactericidal activity, the ability to inhibit bacterial adhesion and quorum sensing, and the capacity to contribute to the restoration of uroepithelium and immune response. Although the emerging findings are promising, only *Arctostaphylos uva ursi* has been employed in human studies (only two) as pre-treatment, with controversial results concerning the subsequent antibiotic use. Few clinical trials have been conducted to evaluate the efficacy of vitamins, alone or in combination, in UTI management. Again, the results obtained are either conflicting, as in the case of vitamin D, or, at most, suggest use in combination treatments, as in the case of vitamin C. Therefore, for the majority of the bioactive compounds reviewed, lack of translation in human clinical studies is a critical limitation for their potential introduction into clinical practice. To broaden the choice of alternative non-antibiotic approaches in UTI management, well-designed clinical trials to investigate the more promising bioactive compounds by in vitro and preclinical studies are warranted.
